# High CCR5 Density on Central Memory CD4^+^ T Cells in Acute HIV-1 Infection Is Mostly Associated with Rapid Disease Progression

**DOI:** 10.1371/journal.pone.0049526

**Published:** 2012-11-21

**Authors:** Xue Yang, Yan-mei Jiao, Rui Wang, Yun-xia Ji, Hong-wei Zhang, Yong-hong Zhang, De-xi Chen, Tong Zhang, Hao Wu

**Affiliations:** Center for Infectious Diseases, Beijing You’an Hospital, Capital Medical University, Beijng, China; University of Cape Town, South Africa

## Abstract

CD4+ central memory T cells play a critical role in the pathogenesis of simian immunodeficiency virus disease, and the CCR5 density on the surface of CD4 T cells is an important factor in human immunodeficiency virus (HIV)-1 disease progression. We hypothesized that quantifying central memory cells and CCR5 expression in the early stages of HIV-infection could provide useful prognostic information. We enrolled two different groups of acute HIV-infected subjects. One group progressed to CD4 T cell numbers below 250 cells/µl within 2 years (CD4 Low group), while the other group maintained CD4 cell counts above 450 cells/µl over 2 years (CD4 High group). We compared the CCR5 levels and percentage of CD4 subsets between the two groups during the 1st year of HIV infection. We found no differences between the two groups regarding the percentage of naïve, central memory and effector memory subsets of CD4 cells during the 1st year of HIV-1 infection. CCR5 levels on CD4+ CM subset was higher in the CD4 Low group compared with the CD4 High group during the 1st year of HIV-1 infection. High CCR5 levels on CD4 central memory cells in acute HIV infection are mostly associated with rapid disease progression. Our data suggest that low CCR5 expression on CD4 central memory cells protects CD4 cells from direct virus infection and favors the preservation of CD4^+^ T cell homeostasis.

## Introduction

Early events during human immunodeficiency virus (HIV) infection are associated with the rate of subsequent disease progression [1], [2]. The identification and measurement of biomarkers correlating to disease progression during early infection would be useful for further understanding HIV pathogenesis and highly valuable for identifying individuals most likely to benefit from early therapeutic intervention.

The entry of HIV into a cell is initiated by the interaction between the virus’s surface envelope proteins and two cell surface components of the target cell, namely CD4 and a chemokine coreceptor, usually CCR5 [3], [4]. Research shows that the surface expression of CCR5 on CD4^+^ and CD8^+^ T cells from AIDS patients is higher than subjects infected with HIV but with no symptoms and healthy controls [5]. The paucity of CD4^+^ CCR5^+^ T cells is a typical feature of natural SIV hosts [6]. The expression of CCR5 positively correlates with viral load, and negatively correlates with CD4+ T cell counts [7]–[9]. This suggests that the expression of CCR5 is closely related to disease progression in HIV infection.

CD4^+^ lymphocytes are the main targets for HIV-1 infection with various sub-populations infected to a different extent [10], [11]. Naïve and memory lymphocyte subsets differ in body distribution, proliferative capacity and expression levels of CCR5 and CXCR4, the main co-receptors for HIV-1 [12]–[17]. CD4^+^ memory T cells can be subdivided into phenotypically distinct subsets with different functions. Central memory (CM) cells, which localize to the blood and secondary lymphoid tissues, are capable of regeneration and long-term maintenance. These can differentiate to effector memory (EM) cells, which are more prevalent in peripheral tissues and provide immediate effector functions at sites of inflammation.

Several studies have shown that CD4 CM cells are the main T cell subset that correlate to the loss or preservation of CD4 cells in HIV or SIV infection [18]–[21]. In the SIV-infected macaque model, the loss of CD4 EM cells is largely driven by the lack of replenishment of CD4 CM cells [22]. In HIV-1 infected humans, preserved CD4 CM cells are strongly associated with the preservation and reconstitution of host immunity [20], [23]. In addition, CD4 CM cells of sooty mangabey that express low amounts of CCR5 showed a reduced susceptibility to SIV infection both *in vivo* and *in vitro* when compared with CD4^+^ CM cells of rhesus macaques [24]. These data suggest that low CCR5 expression on sooty mangabey CD4^+^ T cells favors the preservation of CD4^+^ T cell homeostasis and promotes an AIDS-free status by protecting CD4 CM cells from direct virus infection [24].

**Figure 1:**
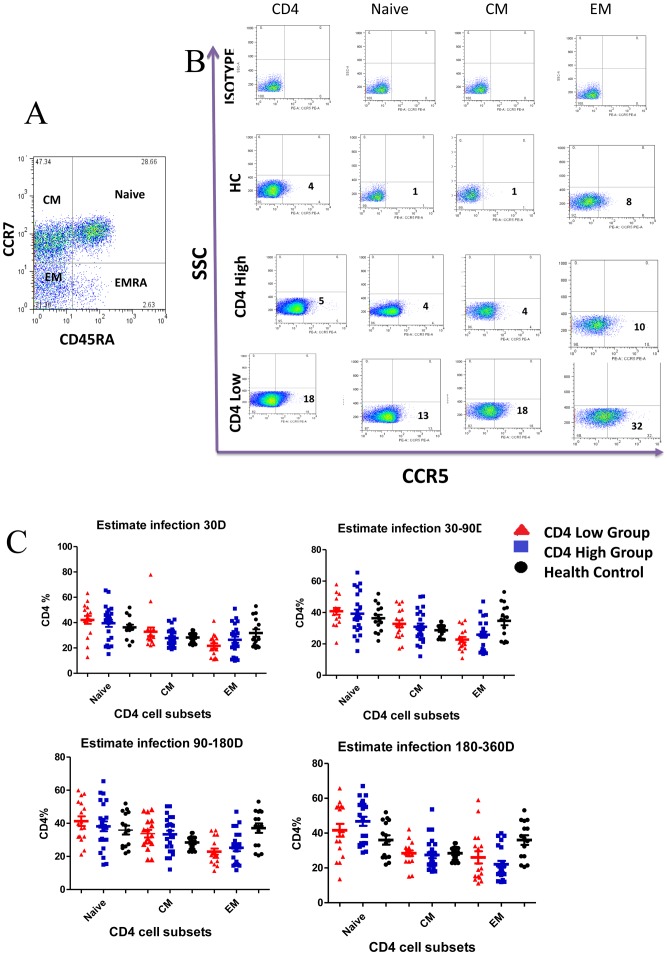
Comparison of the percentage of CD4 subsets between the CD4 High and CD4 Low groups. (A)Based on the expression of CD45RA and CCR7, CD4 T cells were subdivided into naive(CD45RA+CCR7+), central memory(CM; CD45RA–CCR7+) and effect memory (EM; CD45RA–CCR7–) cell subsets. (B–C)Comparison of the percentage of CD4 subsets between the CD4 High and CD4 Low groups during the first year of HIV infection.

In this study, we wanted to evaluate whether CCR5 density and CD4 CM cell quantification in acute HIV infection is associated with rapid disease progression. Two groups of patients with clearly different disease progression were enrolled. Seventeen HIV-patients progressed rapidly and their CD4 counts fell below 250 cells/µl within 2 years (CD4 Low group), while the other 23 patients maintained CD4 counts above 450 cells/µl (CD4 High group). We found no significant difference between the two groups regarding the percentage of naïve, CM and EM subsets of CD4 cells during the 1st year of HIV-1 infection. CCR5 expression on CD4+ CM subset was higher in the CD4 Low group compared with the CD4 High group during the 1st year of HIV-1 infection. High CCR5 levels on CD4 CM cells during acute HIV infection are mostly associated with rapid disease progression.

**Figure 2:**
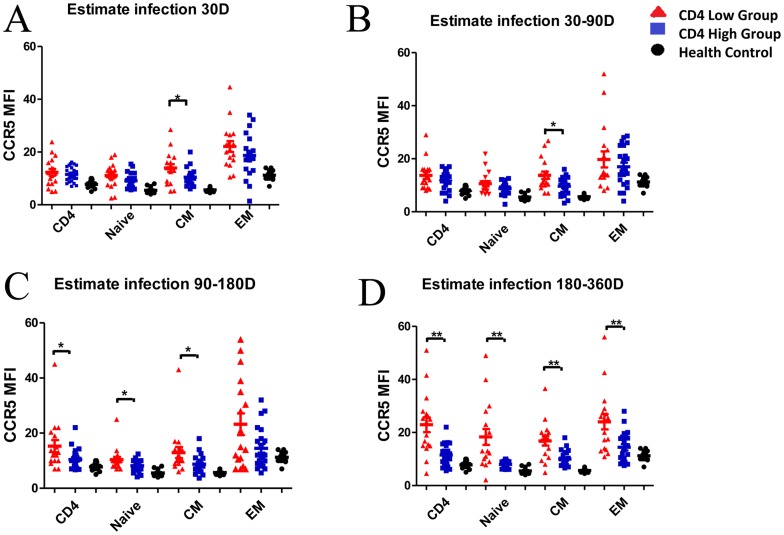
Comparison of the median fluorescence intensity (MFI) of CCR5 on CD4 subsets between the CD4 High and CD4 Low groups. Comparison of the median fluorescence intensity (MFI) of CCR5 on CD4 subsets between the CD4 High and CD4 Low groups among estimated infection 30day(A), 30–90 day(B), 90–180day(C), 180–360day(D). **p<0.01, *p<0.05.

## Results

### No Difference in the Percentage of CD4 Cell Subsets between the CD4 High and CD4 Low Groups

We compared the percentage of naive (CD45RA^+^CCR7^+^), CM (CD45RA^–^CCR7^+^) and EM (CD45RA^–^CCR7^–^) CD4^+^ T cells between the CD4 High and CD4 Low groups during the 1st year of HIV infection (Figure 1A). We found no differences in percentage of the three subsets between the two groups during the 1st year of HIV infection (Figure 1C).

**Figure 3:**
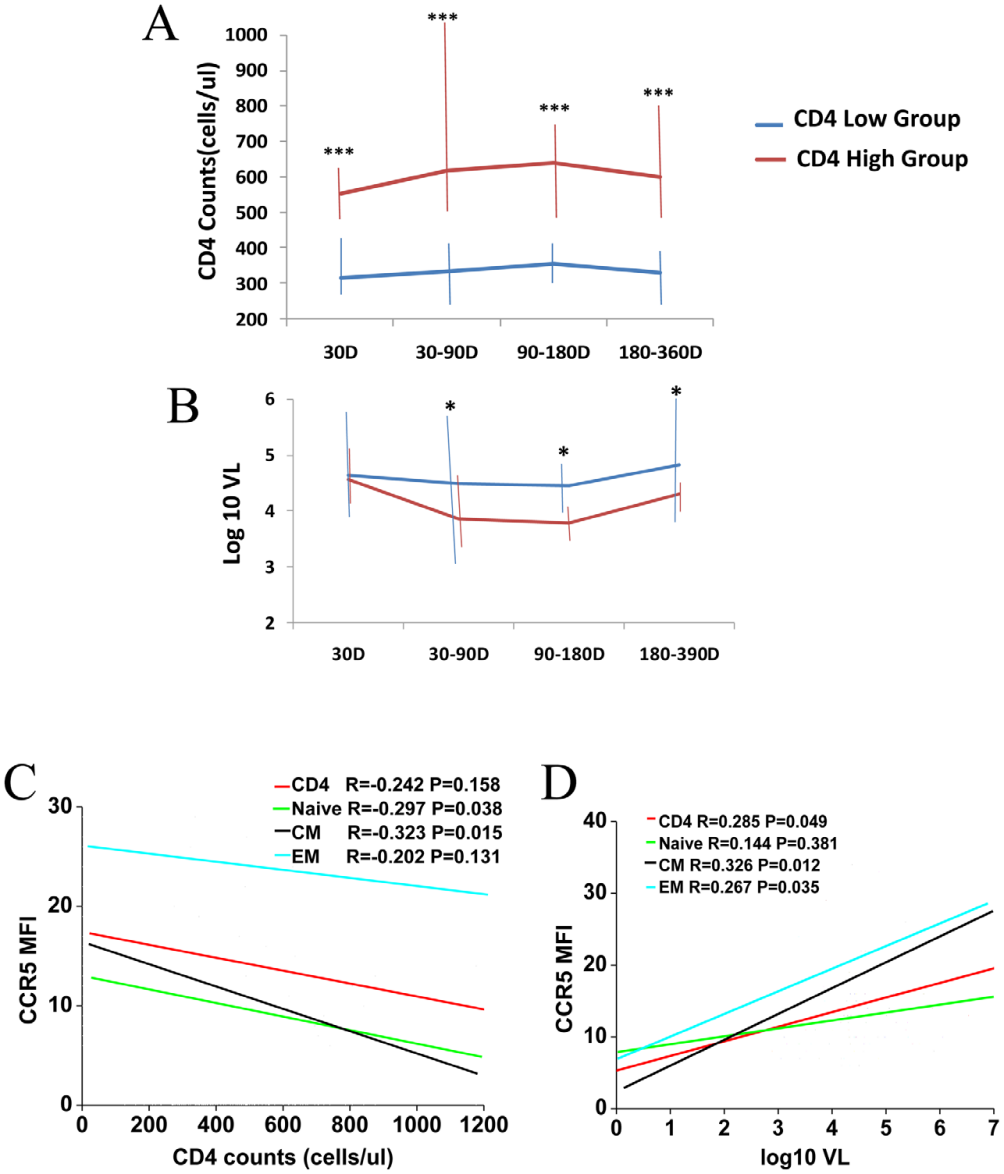
Analysis the correlation between CCR5 MFI on CD4 subsets and disease progression. Compare the CD4(A) and viral load(B) between the CD4 high and CD4 low groups. The correlations of CCR5 MFI on CD4 subsets with CD4+T cells(C) and viral load (D). *p<0.05,**p<0.01,,***p<0.001 Data are expressed as the average and range for Figure 3A and B.

**Figure 4:**
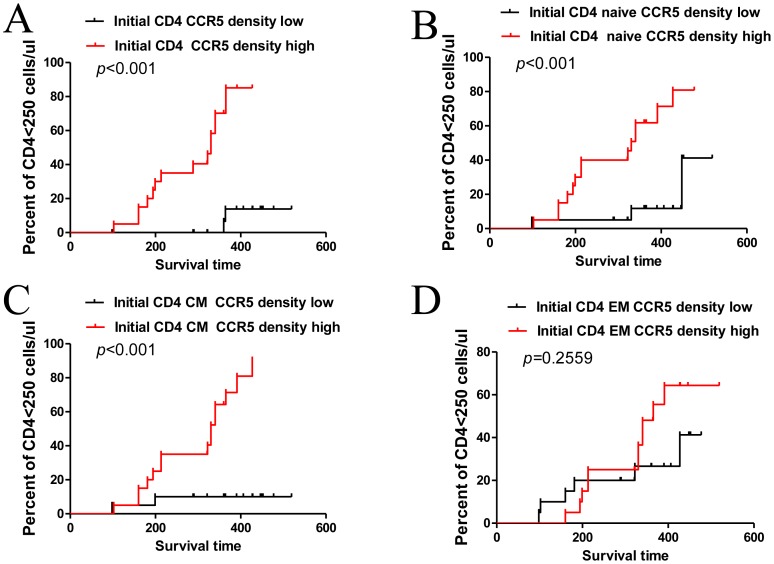
Analysis the association between initial CCR5 expression on CD4 subsets and the time for CD4<250 cells/ul. Initial CCR5 expression on CD4(A), naïve(B) and CM(C) were associated with the time taken for the study participants’ CD4 counts to fall below 250 cells/µl. There was no obvious association between initial CCR5 expression on EM with the time taken for the study participants’ CD4 counts to fall below 250 cells/µl.

### High Levels of CCR5 Expression on CD4+ CM Cells in the CD4 Low Group

We compared CCR5 levels on total CD4, naive, CM and EM CD4+ cells between the CD4 High group and CD4 Low group during the 1st year of HIV infection. We found that CCR5 level was significantly lower only on CM subset from the CD4 High group when compared with the CD4 Low group within the first 90 days of HIV infection (Figure 2A,2B). With the disease progression, CCR5 level was significantly lower on all subsets from the CD4 High group when compared with the CD4 Low group(Figure 2C,2D). Of note, CD4+ EM cells in both groups contained a higher fraction of CCR5+ cells compared with CM cells, with the lowest levels of CCR5 expression being present on naïve cells during the 1st year of HIV infection (Figure 2A–D).

**Table 1:** Characteristics of subjects in this study.

patients	Cases	Age	Initial Fiebig stage	InitialCD4 counts (cells/ul)	last CD4 counts(cells/ul)	Initial VL (copies/ml)	VL set point (copies/ml)	Days from the initial Positive point to CD4<250 cells/ul
Total	40	30.3 (19–52)	III–V	468.92 (271–637)	340.53 (129–653)	143,000 (1,610–768,000)	44,000 (14,600–576,000)	NA
CD4 high	23	30.5 (19–46)	III–V	547.62 (492–637)	485.74 (450–653)	33,500 (1,610–235,000)	27,500 (14,600–161,000)	NA
CD4 low	17	30 (23–52)	IV–V	366.54 (271–450)	171.11 (127–249)	692,000 (48,200–768,000)	109,000 (40,300–576,000)	196
HC	15	30 (22–44)		753 (540–1,200)				

NA: not applicable; HC: healthy control; VL: viral load.

### CCR5 Expression on CM Cells is Positively Associated with Viral Load and Negatively Associated with CD4 Cell Counts

We also compared the CD4 and viral load between the two groups during the first year of HIV infection. We found that CD4 was significantly lower in CD4 low group compared with CD4 high group during the first year of HIV infection(Figure 3A). The viral load was no difference between the two groups during the first 30 days of HIV infection(Figure3B). With the disease progression, the viral load of the CD4 low group is higher than that of the CD4 high group(Figure3B). Next, the relationship between CCR5 density on CD4 subsets (naïve, CM and EM) and viral load and CD4 counts was analyzed. We found that CCR5 expression on CM and naïve was negatively associated with CD4 counts (r = −0.323, p = 0.015 for CM; r = −0.297, p = 0.038 for naive; Figure 3C). There was no correlation between CCR5 expression on EM cells and CD4 counts (r = −0.202, p = 0.131, Figure 3C). CCR5 expression on CM and EM cells was positively associated with viral load (r = 0.326, p = 0.012 for CM; r = 0.267, p = 0.035 for EM; Figure 3D) but there was no correlation between CCR5 expression on naive cells and viral load (r = 0.144, p = 0.381, Figure 3D). CM is the only subset that CCR5 expression on this subset are both positively associated with viral load and negatively associated with CD4 counts.

### Early High CCR5 Expression on Naïve and CM Subsets are Associated with a Reduction in CD4 Counts below 250 Cells/µl

Next, we analyzed the association of the initial CCR5 expression on CD4 subsets (naïve, CM and EM) with the time taken for CD4 counts to fall below 250 cells/µl using the Log-rank (Mantel-Cox) test. We found that high levels of CCR5 on CM and naïve subsets, but not EM cells, were associated with the time taken for CD4 counts to fall below 250 cells/µl (Figure 4B, C, D).

## Discussion

The aim of this study was to identify correlates of disease progression measured during early HIV infection. In the present study, we compared CD4+ cell subsets (naïve, CM and EM) and CCR5 expression levels in the 1st year of HIV infection between two distinct groups of patients. One group progressed to CD4 cell numbers below 250 cells/µl within 2 years, while the other group maintained CD4 cell counts above 450 cells/µl within 2 years.

Studies using the SIV model of infection have demonstrated the critical role of CD4+ CM cells in the pathogenesis of HIV disease. In SIV-infected macaques the ultimate loss of CD4 EM cells is in large part driven by lack of replenishment of this compartment by CD4 CM cells [18]. Other studies have shown that vaccinated macaques survive longer when CD4 CM cell numbers are preserved [19] and that HIV-1 viral controllers tend to preserve CD4 CM cell numbers [20]. However, in this study we did not observe a percentage difference in the CD4 subsets (naïve, CM and EM) between the CD4 High and CD4 Low groups during the 1st year of HIV infection. Remodeling of the memory compartment may be sufficiently variable during early infection such that memory cells may not provide a useful predictor for disease progression. Our findings are consistent with a previous study that reported a lack of association between CM cells and disease progression in early HIV infection [1]. Thus, CM cell numbers may not provide a predictor for disease progression in early HIV infection. It has been reported that the density of CCR5 on the surface of CD4 T cells is an important factor in HIV-1 disease progression [7], [9]. Low levels of SIV infection in sooty mangabey CM CD4^+^ T cells are associated with limited CCR5 expression [24]. Thus, it would be useful to determine whether high CCR5 expression on CM cells in early HIV infection is associated with rapid disease progression. We found that high CCR5 expression only on CM subset significantly higher in the CD4 Low group compared with the CD4 High group within the first 90 days of HIV infection. We also observed that CCR5 expression on CM cells was mostly associated with disease progression compared with CCR5 expression on naïve and EM cells. The high CCR5 expression on CD4 CM cells may be the result of a high HIV infection rate of CD4 CM cells. A progressive depletion of CM CD4^+^ T cells from the peripheral blood was observed, accompanied by high levels of viral replication in the cells of this subtype [25]. We propose that in the CD4 High group, low CCR5 expression on CD4 CM cells protects against virus-mediated depletion and thus favors the preservation of CD4^+^ T cell homeostasis. Thus, it may be useful to down modulate CCR5 expression on CM cells in early HIV infection to delay disease progression.

In conclusion, we found that a high CCR5 density on CM CD4^+^ T cells in acute HIV-1 infection is mostly associated with rapid disease progression. As we had no specimens available before HIV infection, it is important to determine whether the difference in CCR5 expression on CD4 subsets is inherent or HIV-induced.

## Patients and Methods

### Patients

Forty patients recently infected with HIV-1 (from Fiebig stage III to Fiebig stage V) from a group of HIV-1-negative high-risk MSM (men who have sex with men) cohorts who were screened every 2 months for HIV-1 infection in Beijing You’an Hospital were enrolled. The patients were divided into two groups with significant disease progression: one group of 17 patients (CD4 Low group) progressed to CD4 counts <250 cells/µl within 2 years, while the other group (CD4 High group) of 23 patients maintained CD4 counts higher than 450 cells/µl. The progression of early HIV-1 infection can be depicted in six discrete stages proposed by Fiebig et al. [26], [27]. The infection time is estimated according to Fiebig stages [26], [27]. The characteristics of patients in this study are presented in Table 1. Samples were collected at the first positive point and 1, 2, 4, 8, 12, 24, 36, 48, 60, 72, 84, 96, and 108 weeks after the first positive point. 15 health persons whose gender and age are match with the HIV patients were enrolled as control. This study was approved by the Ethical Committee at Beijing You’an Hospital and written informed consent was obtained from all participants.

### Flow Cytometric Analysis

The monoclonal antibodies (mAbs) CD4-PerCP and CD45RA-FITC were purchased from BD Bioscience (San Diego, CA, USA). CCR7-APC and CCR5-PE were purchased from eBioscience (San Diego, CA, USA). Based on the expression of CD45RA and CCR7, CD4 T cells were subdivided into naive, CM and EM cell subsets (CD4^+^ Naïve, CD4^+^ CM and CD4^+^ EM, respectively) as follows: naive cells (CD45RA^+^CCR7^+^); CM cells (CD45RA^−^CCR7^+^); EM cells (CD45RA^−^CCR7^−^). To investigate the change of CD4^+^ subset numbers and the expression of CCR5, freshly isolated peripheral blood mononuclear cells were incubated with CD4-PerCP, CD45RA-FITC, CCR5-PE and CCR7-APC for 20 min at 4°C. After incubation with the various antibodies, the cells were washed twice with phosphate-buffered saline (1 × PBS). Four-color flow cytometric analyses were then performed using FACS Calibur and CELL Quest software (Becton Dickinson, San Jose, CA).

### Assays for CD4 T Cell Counts and Plasma HIV-1 RNA

After whole-blood lysis (FACSlysing Solution, Becton Dickinson San Diego, CA, USA), T lymphocyte counts were determined by three-color flow cytometry using CD3-APC, CD4-FITC and CD8-PE monoclonal antibodies (BD Bioscience San Diego, CA, USA). The analysis was performed using a BD FACSCount flow cytometer in accordance with Chinese Center for Disease Control and Prevention guidelines.

Plasma HIV RNA was quantified by real-time PCR (Roche, Germany), a super-sensitive method. The sensitivity of detection of this assay was 40 copies/ml.

### Statistical Analyses

All data were analyzed using SPSS version 16.0 for Windows software (SPSS Inc, Chicago, IL). Correlation between two parameters was determined using Spearman’s correlation coefficient. Non-parametric Mann-Whitney *U*-test was used to compare medians between the groups. Log-rank (Mantel-Cox) test was used to compare the survival. A value of p<0.05 was considered as statistically significant.
